# Deposition of hydrophilic Ti_3_C_2_T_x_ on a superhydrophobic ZnO nanorod array for improved surface-enhanced raman scattering performance

**DOI:** 10.1186/s12951-022-01756-4

**Published:** 2023-01-16

**Authors:** Zhihua Wu, De Zhao, Xin Han, Jichang Liu, Ying Sun, Yaogang Li, Yourong Duan

**Affiliations:** 1 State Key Laboratory of Oncogenes and Related Genes, Shanghai Cancer Institute, Renji Hospital School of Medicine, Shanghai Jiao Tong University, Shanghai, 200032 China; 2 State Key Laboratory for Modification of Chemical Fibers and Polymer Materials, International Joint Laboratory for Advanced Fiber and Low-Dimension Materials, College of Materials Science and Engineering, Donghua University, Shanghai, 201620 China; 3 State Key Laboratory of Chemical Engineering, School of Chemical Engineering, East China University of Science and Technology, Shanghai, 200237 China

**Keywords:** SERS, Superhydrophobic substrate, Ti_3_C_2_T_x_, DFT calculations, miRNA detection

## Abstract

**Background:**

Superhydrophobic substrate modifications are an effective way to improve SERS sensitivity by concentrating analyte molecules into a small surface area. However, it is difficult to manipulate low-volume liquid droplets on superhydrophobic substrates.

**Results:**

To overcome this limitation, we deposited a hydrophilic Ti_3_C_2_T_x_ film on a superhydrophobic ZnO nanorod array to create a SERS substrate with improved analyte affinity. Combined with its interfacial charge transfer properties, this enabled a rhodamine 6G detection limit of 10^−11^ M to be achieved. In addition, the new SERS substrate showed potential for detection of biological macromolecules, such as microRNA.

**Conclusion:**

Combined with its facile preparation, the SERS activity of ZnO/Ti_3_C_2_T_x_ suggests it may provide an ultrasensitive environmental pollutant-monitoring and effective substrate for biological analyte detection.

**Graphical Abstract:**

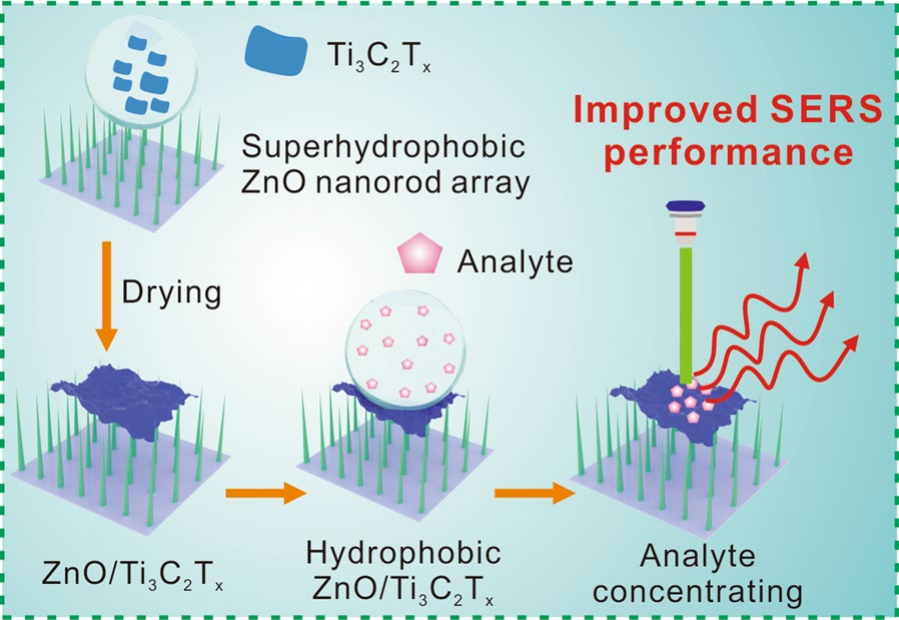

**Supplementary Information:**

The online version contains supplementary material available at 10.1186/s12951-022-01756-4.

## Introduction

Surface-enhanced Raman scattering (SERS) is a widely used and powerful tool for analyzing molecules and biomarkers [1], in vivo imaging [2], and environmental pollutants [3–6]. Recent development of new surface-enhancing materials and methods has markedly improved SERS detection limits [7–9]. Notably, the combination of nanostructured plasmics and Raman spectroscopy has enabled single molecule detection [10–12]. However, the surface enhancement is restricted to “hot spots” with an area of a few nm^2^ [13–16]. The detection or identification of low-concentration analytes remains challenging because random diffusion of molecules on the hydrophilic substrate results in poor spatial localization [17]. This random diffusion makes it time-consuming to search for molecules of interest within the enhanced area [18, 19].

Various strategies have been used to overcome the above problems [20–22], such as the use of superhydrophobic substrates to markedly enhance SERS intensity [17, 23–26]. Such substrates overcome the diffusion limit by concentrating molecules into a small area, resulting in a threefold increase in Raman intensity compared with ordinary hydrophilic substrates [23]. Superhydrophobic substrates can be fabricated using photolithography and those noble metal nanoparticle decorated SERS platform can achieve a detection limit of 10^−18^ M using rhodamine 6G (R6G) as a SERS probe [17]. However, the fabrication processes are sophisticated and the required equipment is expensive. Thus, more facile methods are required to prepare SERS substrates that confine the analyte molecule. Manipulating the morphology of inorganic nanoarrays may lead to new superhydrophobic substrates [27]. Among these inorganic materials, ZnO is an ideal candidate because it enables the facile synthesis and regulation of nanostructures [28]. Moreover, it can be easily decorated on the surface of different substrates, such as glass slides [29] and polydimethylsiloxane [30]. Although superhydrophobic SERS substrates can increase the Raman intensity remarkably, their poor affinity for aqueous analytes makes these molecules difficult to attach, which reduces the efficiency of the detection process.

**Fig. 1:**
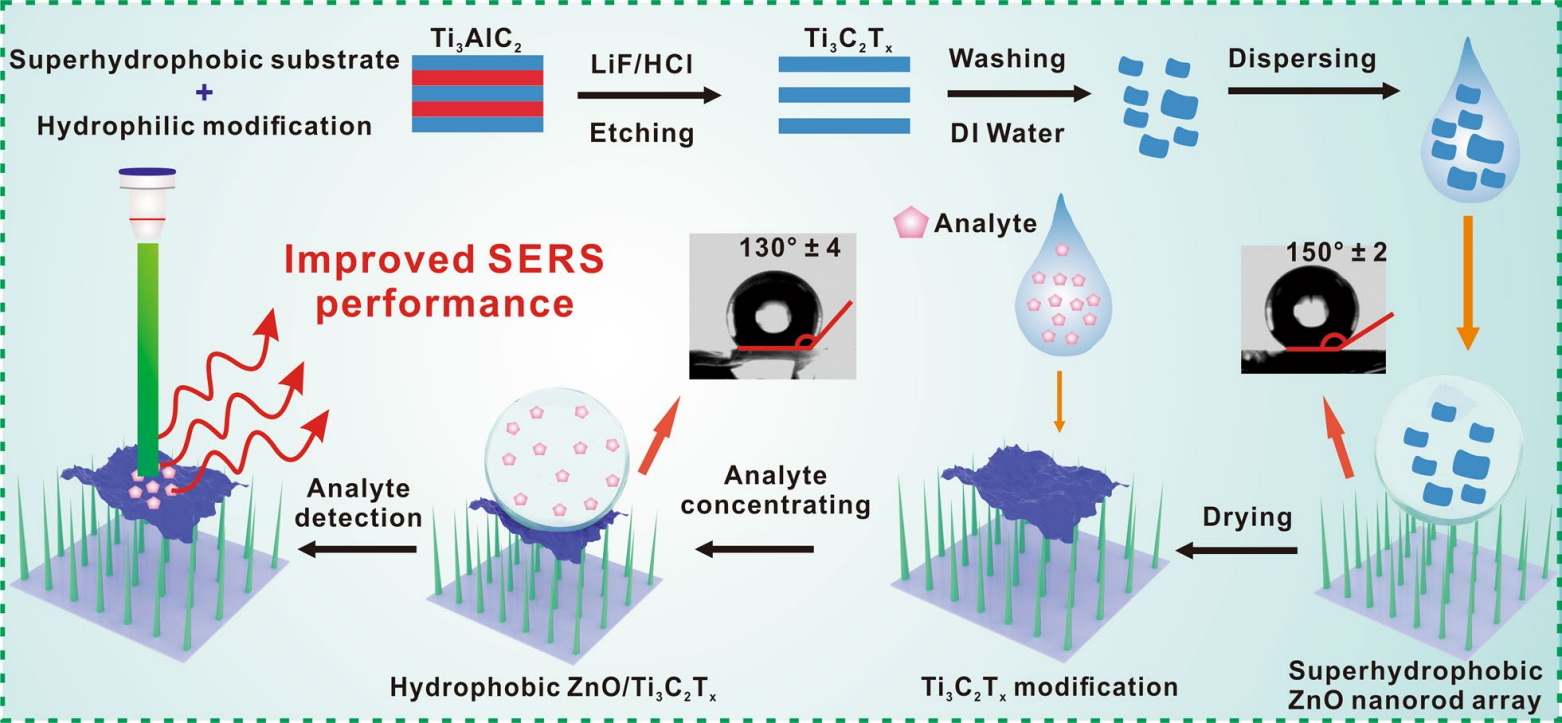
Illustration of the fabrication of a ZnO/Ti_3_C_2_T_x_ substrate and the mechanism of its surface-enhanced Raman scattering (SERS) activity. The Ti_3_C_2_T_x_ monolayer is prepared using a selective etching method and then dispersed in deionized water. The ZnO/Ti_3_C_2_T_x_ substrate is fabricated by depositing the Ti_3_C_2_T_x_ solution on a superhydrophobic ZnO nanorod array

Herein, we report a facile method to fabricate a SERS substrate comprising a two-dimensional (2D) Ti_3_C_2_T_x_ monolayer on a supporting superhydrophobic ZnO nanorod array (Fig. 1). The hydrophilicity of the Ti_3_C_2_T_x_ surface increases the affinity of aqueous analytes and its intrinsic metallic conductivity and plasmon resonance enhance Raman scattering intensity [31–33]. We prepared a small-area Ti_3_C_2_T_x_ spot on a superhydrophobic ZnO nanorod array and demonstrated that the hydrophobic substrate concentrates the analyte within the enhancement area and thus greatly improves the Raman scattering intensity. Density functional theory (DFT) calculations further showed that the interfacial charge transfer also contributes to the intensity enhancement. The ZnO/Ti_3_C_2_T_x_ substrate provides a cost-effective SERS platform for ultrasensitive analyte detection and identification.

## Material and methods

### Material

LiF was purchased from Aladdin Reagents (Shanghai), 200 mesh MAX Ti_3_AlC_2_ was purchased from 11 technology co.,LTD, hydrochloric acid (HCl), Zinc nitrate hexahydrate (Zn(NO_3_)_2_), Hexamethylenetetramine (HMTA), potassium permanganate were purchased from Sinopharm Chemical Reagent Co. Ltd. (China), Rhodamine 6G (R6G), methylene blue (MB), acid blue (AB), crystal violet (CV) were purchased from MACKLIN. All chemicals were used as received without further purification. miRNA with sequence of UUUGUACUACACAAAAGUACUG (sense(5'-3')) was purchased from RIBOBIO co.,LTD.

### Fabrication of monolayer Ti_3_C_2_T_x_

Monolayer Ti_3_C_2_T_x_ was obtained as previously described [34, 35]. Typically, 1.6 g LiF was added in 20 mL 9 M HCl in 5 min and magnetic stirred for another 5 min. Then, 1 g Ti_3_AlC_2_ was added over the course of 5 min in case of overheat. Etching was performed at room temperature for 24 h. The acidic mixture was centrifugated at 3500 rpm until pH ≥ 5 (each cycle for 5 min). Sediment was re-dispersed by hand-shaking every time. The Ti_3_C_2_T_x_ colloid was collected when the supernatant turned stable dark-green. After that, monolayer Ti_3_C_2_T_x_ was collected after centrifugation at 3500 rpm for 1 h by collecting the supernatant.

### Preparation of ZnO/Ti_3_C_2_T_x_ SERS substrate and enhancement performance

Glass slides were pretreated using potassium permanganate and n-butanol as described previously [36]. To successfully construct ZnO nanorods on the glass slide surface, the pretreated glasses were firstly encapsulated by PDMS to form a 75 mm × 4 mm × 1 mm microchannel. Then, reaction solutions of 0.05 M Zn(NO_3_)_2_ and 0.05 M HMTA were injected by pump in two Teflon tubes at 50 µL/min for 2 h at 90 ℃ [37, 38]. After that, PDMS was removed and the obtained ZnO decorated glass slide was thoroughly washed by distilled water. The ZnO decorated glass slide was then dried in oven at 90 ℃ for further use. ZnO film was prepaered as previously described[39].

To fabricate ZnO/Ti_3_C_2_T_x_ SERS substrate, 4 µL 1–2 mg/mL monolayer Ti_3_C_2_T_x_ aqueous solution was dropped onto ZnO decorated glass slide and dried at room temperature for further use. Before test, 4 µL 10^–6^ to 10^–10^ M R6G was dropped on the ZnO/Ti_3_C_2_T_x_ SERS substrate and dried at room temperature. Laser of 532 nm was used in the experiments at power of 5 mW with a static mode and 20 s exposure time to reduce background noise. As for Raman mapping, step of 1 µm and 2 s exposure time at 532 nm were adopted.

### DFT simulations and calculations

First-principles calculations of the enhancing mechanism in our systems used the DFT-based projector augmented-wave method implemented in the Materials Studio software [40]. The exchange–correlation energy was treated using the generalized gradient approximation of Perdew, Burke, and Ernzerhof [41, 42]. The energy cutoff for the plane wave basis expansion was set to 400 eV during the geometry relaxation. The 3 × 3 × 1 Monkhorst–Pack k-point sampling grid was used to calculate the surface structures. The self-consistent calculations used an energy convergence threshold of 10^−5^ eV. The equilibrium lattice constants were optimized using a maximum stress constraint of 0.03 eV/Å on each atom. Spin polarization was allowed in all calculations. The Ti_3_C_2_ (001) and ZnO (101) surfaces were obtained from their corresponding bulk supercell structures. To describe the charge transfer at the interface of these structures, lattice parameters of *a* = 12.384727 and *b* = 19.073 were established. Finally, the molecular adsorption energy (*E*_b_) was calculated using the expression [43] *E*_b_ = *E*_total_ − *E*_1_ − *E*_2_, where *E*_1_, *E*_2_, and *E*_total_ are the respective energies of the interface, molecule, and interface with molecules adsorbed.

## Results and discussion

### Characterization of Ti_3_C_2_T_x_ nanosheets

**Fig. 2:**
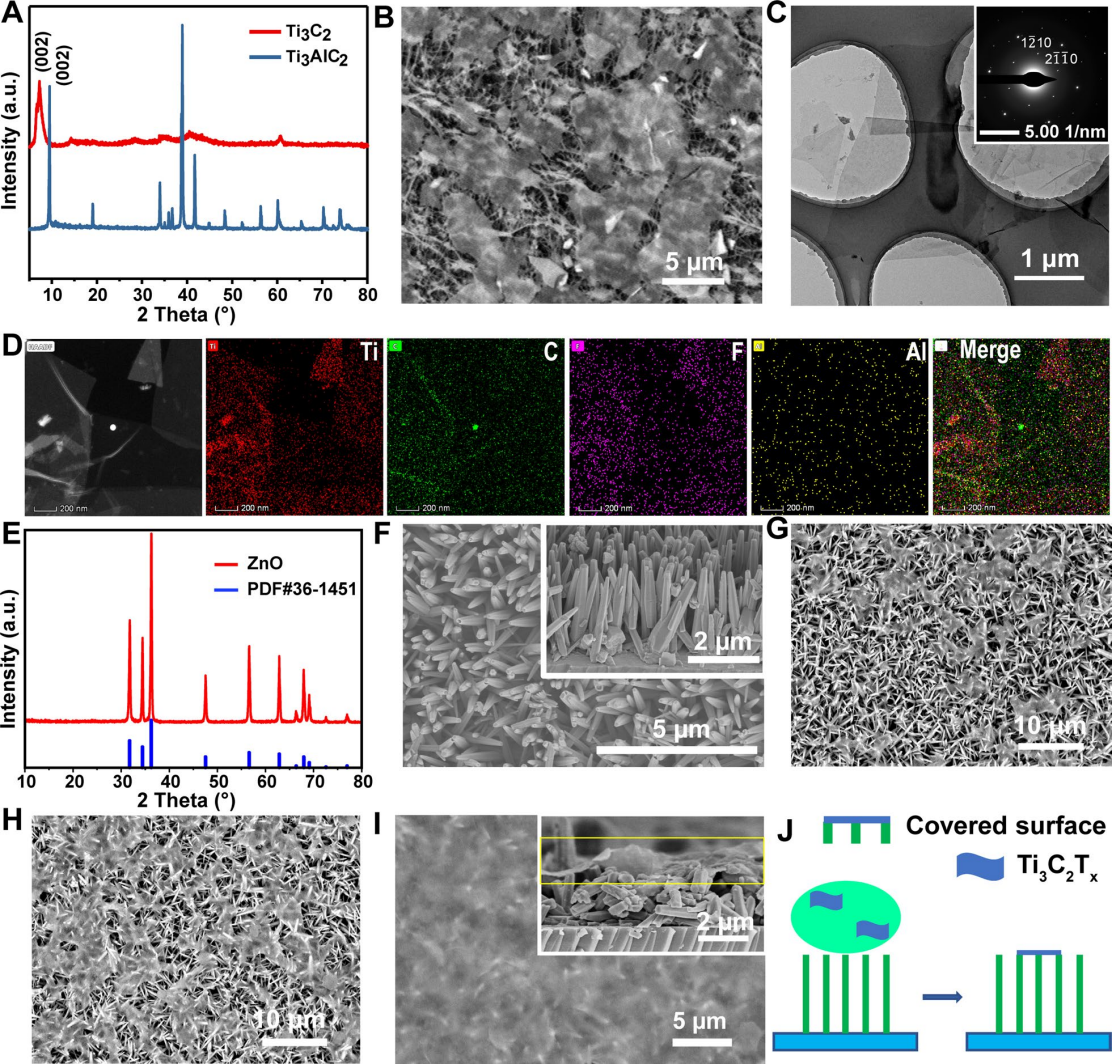
**A** X-ray diffraction (XRD) patterns of pristine Ti_3_AlC_2_ and Ti_3_C_2_T_x_. **B** Scanning electron microscopy (SEM) image of a Ti_3_C_2_T_x_ monolayer under low magnification. **C** High-resolution transmission electron microscopy image of a Ti_3_C_2_T_x_ nanosheet and the corresponding selected area electron diffraction pattern. **D** Scanning TEM (STEM) coupled with energy dispersive X‐ray spectroscopy (EDX) of a Ti_3_C_2_T_x_ nanosheet. **E** XRD patterns of ZnO nanorod arrays with hexagonal wurtzite structure (Joint Committee on Powder Diffraction Standards card no. 36–1451). **F** SEM image of the ZnO nanorod array without Ti_3_C_2_T_x_, and SEM images after drop-casting 4 µL of **G** 1 mg/mL, **H** 1.5 mg/mL, and **I** 2 mg/mL Ti_3_C_2_T_x_ solutions on the ZnO nanorod array, the inset is a sectional view of ZnO/Ti_3_C_2_T_x_. **J** Illustration of the ZnO/Ti_3_C_2_T_x_ substrate fabrication process

Two-dimensional Ti_3_C_2_T_x_ nanosheets were prepared by selectively etching the Al layer from Ti_3_AlC_2_. As shown by the X-ray powder diffraction (XRD) patterns (Fig. 2A), the main peaks corresponding to MAX-phase Ti_3_AlC_2_ disappeared after etching, and the (002) diffraction angle decreased from 9.5° to 7.5°. Large Ti_3_C_2_T_x_ nanosheets were obtained using an in situ HCl/LiF etching method (Fig. 2B). High-resolution transmission electron microscopy and selected area electron diffraction showed that monolayered Ti_3_C_2_T_x_ sheets were successfully derived from MAX-phase Ti_3_AlC_2_ (Fig. 2C). Elemental composition maps obtained using X-ray energy-dispersive spectroscopy further proved that the Al layer was completely removed from the Ti_3_C_2_T_x_ surface (Fig. 2D, Additional file 1: Figure S1). These results demonstrate the successful etching of the atomic Al layer in Ti_3_AlC_2_.

### Characterization of the ZnO/Ti_3_C_2_T_x_ SERS substrate

The ZnO nanorod arrays were prepared using a microfluidic chemical reaction. As shown in Fig. 2E, the XRD spectrum of the prepared ZnO was the same as that of ZnO with hexagonal wurtzite structure. Field emission SEM showed that the inclined ZnO nanorods with a length of ≈2.5 µm and a uniform hexagonal structure were successfully decorated on the glass substrate (Fig. 2F).

Small-area ZnO/Ti_3_C_2_T_x_ spots were prepared by depositing Ti_3_C_2_T_x_ solutions on a ZnO nanorod-modified glass substrate. To prevent oxidation of the Ti_3_C_2_T_x_, the spots were dried at room temperature. As shown in Fig. 2G–I, the coverage area of the 2D Ti_3_C_2_T_x_ nanosheets was improved by increasing the concentration of the Ti_3_C_2_T_x_ solution. Furthermore, Fig. 2H shows that the Ti_3_C_2_T_x_ nanosheets were supported by the tips of ZnO nanorods and closely attached to the inclined nanorods. With the increasing concentration of Ti_3_C_2_T_x_, a thin film gradually formed on the ZnO nanorod array (inset of Fig. 2I). Therefore, ZnO/Ti_3_C_2_T_x_ was fabricated by concentrating Ti_3_C_2_T_x_ nanosheets on ZnO nanorod arrays surface (Fig. 2J).

### Hydrophilicity of prepared substrates

**Fig. 3:**
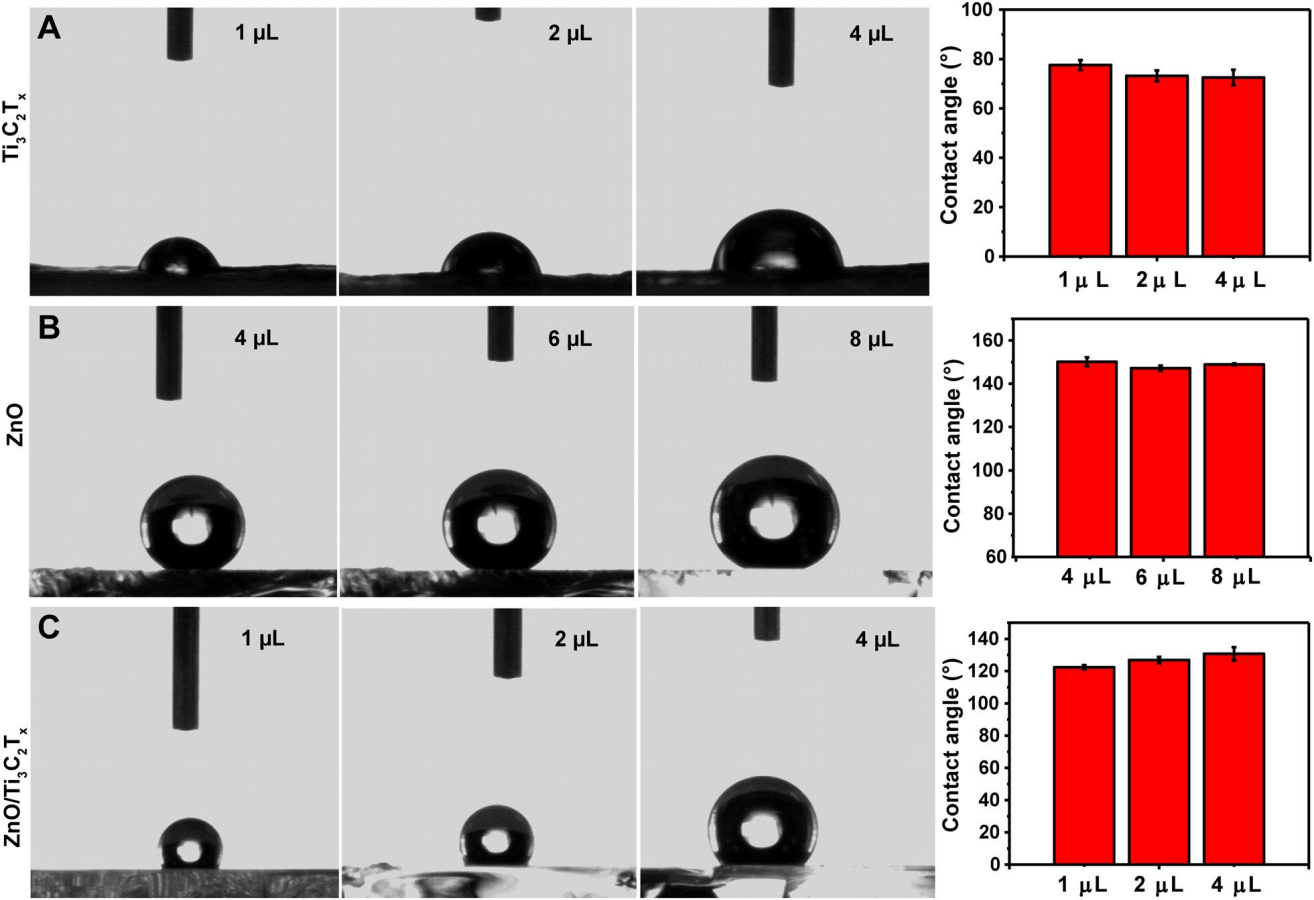
Characterization of water droplet contact angles on **A** pristine Ti_3_C_2_T_x_ film, **B** the ZnO nanorod array, and **C** ZnO/Ti_3_C_2_T_x_

The hydrophilicities of the Ti_3_C_2_T_x_ film, ZnO nanorod array, and ZnO/Ti_3_C_2_T_x_ were assessed by measuring the surface contact angles of water droplets. The Ti_3_C_2_T_x_ film was hydrophilic, having contact angles of 77.6 ± 2° and 72.5 ± 3° for droplet volumes of 1 and 4 μL, respectively (Fig. 3A). The ZnO nanorod array surface was superhydrophobic (Fig. 3B), having a low affinity for droplet volumes less than 4 µL. Even 4 μL droplets maintained a contact angle of 150.2 ± 2° and a relatively spherical shape. Under gravity, droplet volumes of 6 and 8 µL made greater contact with the substrate surface and had an ellipsoidal shape. These results proved that the superhydrophobic substrate was successfully fabricated by decorating the glass slide with the ZnO nanorod array. Coating the ZnO with Ti_3_C_2_T_x_ resulted in a contact angle of 130.7 ± 4° for 4 µL droplets (Fig. 3C). Smaller contact angles were observed for droplet sizes less than 4 µL, which may be explained by the micro/nanostructure of ZnO/Ti_3_C_2_T_x_. For example, if the droplet is larger than the hydrophilic modification area, the edge of ZnO/Ti_3_C_2_T_x_ substrate can still be superhydrophobic. Importantly, ZnO/Ti_3_C_2_T_x_ showed a higher hydrophilicity than the ZnO nanorod array, as evidenced by the smaller contact angles and easier droplet attachment. These results demonstrate that the ZnO/Ti_3_C_2_T_x_ substrate greatly improved the water droplet attachment and maintained high contact angles after Ti_3_C_2_T_x_ modification.

### SERS performance of the ZnO/Ti_3_C_2_T_x_ substrate

**Fig. 4:**
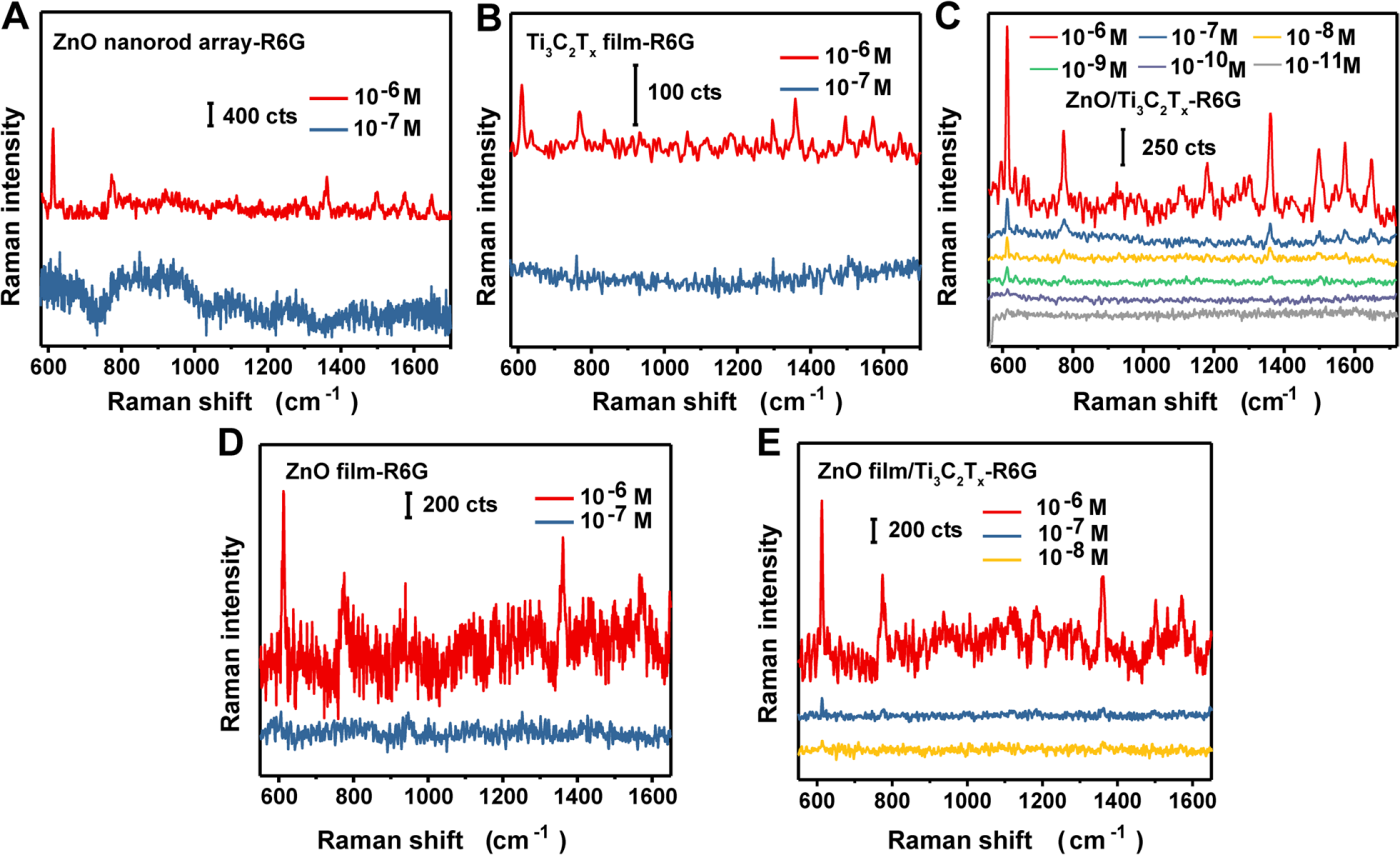
SERS performance of **A** the superhydrophobic ZnO nanorod array, **B** Ti_3_C_2_T_x_ film, **C** ZnO/Ti_3_C_2_T_x_ (R6G analyte concentration ranging from 10^−6^ to 10^−11^ M), and **D** ZnO film, **E** ZnO(film)/Ti_3_C_2_T_x_ (R6G analyte concentration ranging from 10^−6^ to 10^−8^ M)

Amorphous ZnO nanocages have remarkable SERS activity [44]. Here, crystalline ZnO also showed SERS activity, with a hexagonal wurtzite ZnO nanorod array yielding an R6G detection limit of 10^−6^ M. As indicated in Fig. 4A, characteristic peaks of R6G can be clearly identified. However, the characteristic Raman scattering peaks at 614, 773, 1360, 1500, 1573, and 1650 cm^−1^ were almost undetectable at an R6G concentration of 10^−7^ M.

An R6G detection limit of 10^−7^ M was achieved using Ti_3_C_2_T_x_ film (Fig. 4B), which is consistent with a previously reported value [32]. Different Ti_3_C_2_T_x_ films were prepared by filtering Ti_3_C_2_T_x_ nanosheets (0.5–2.0 mg in 30 mL water) on a polytetrafluoroethylene membrane (50 mm of diameter). At an R6G concentration of 10^−6^ M, all films had slight difference of SERS activity (Additional file 1: Figure S2). However, reducing the R6G concentration from 10^−6^ to 10^−7^ M resulted in markedly lower intensities of the Raman peaks at 773 and 1500 cm^−1^ and rendered the characteristic peaks at 614, 1360, 1573, and 1650 cm^−1^ undetectable. The SERS activity of the ZnO/Ti_3_C_2_T_x_ substrate was much higher than that of pristine ZnO and Ti_3_C_2_T_x_, resulting in an R6G detection limit of 10^−11^ M (Fig. 4C) with enhancement factors (EFs) of 1.49 × 10^7^ and 7.88 × 10^6^ at peaks of 614 and 1360 cm^−1^, respectively (Additional file 1: Table S1, calculation details were presented in supporting information). The EF of ZnO/Ti_3_C_2_T_x_ is 2.10fold of Ti_3_C_2_T_x_ and 2.73fold of ZnO at peak of 614 cm^−1^. At 1360 cm^−1^, the EF of ZnO/Ti_3_C_2_T_x_ is 1.65fold and 2.64fold of Ti_3_C_2_T_x_ and ZnO, respectively. As can be seen in Additional file 1: Figure S6, with the decrease of analyte concentration, the distribution of Raman signal becomes uneven. This indicates that the distribution of molecules on the substrate is not uniform with decreasing concentration. According to the Raman mapping, 10^–11^ M R6G could be detected by mapping method. Thus, the combination of ZnO and Ti_3_C_2_T_x_ enhanced the R6G detection sensitivity by five orders of magnitude compared with that of pristine ZnO and four orders of magnitude compared with that of pristine Ti_3_C_2_T_x_. The ZnO/Ti_3_C_2_T_x_ substrate was also highly sensitive to methylene blue (MB), yielding a detection limit of 10^−10^ M (Additional file 1: Figure S7a).

The SERS performance of the ZnO substrate may be influenced by the ZnO/Ti_3_C_2_T_x_ interfacial properties. A ZnO film and a ZnO(film)/Ti_3_C_2_T_x_ substrate were fabricated to assess the contribution of hydrophobic micro/nanostructure to the SERS activity (Additional file 1: Figure S3a and b). The ZnO film substrate had a higher SERS sensitivity compared with that of pristine ZnO, enabling detection of 10^−6^ M R6G (Fig. 4D). The ZnO(film)/Ti_3_C_2_T_x_ substrate had an even higher SERS sensitivity, achieving an R6G detection limit of 10^−8^ M (Fig. 4E). Contact angle measurements indicated both ZnO film and ZnO(film)/Ti_3_C_2_T_x_ were hydrophilic, displaying 4 µL droplet contact angles of 37.1 ± 1.2° and 77.5 ± 1°, respectively (Additional file 1: Figure S4a and b). The above results demonstrate that the hydrophobic micro/nanostructure of ZnO/Ti_3_C_2_T_x_ may contribute more to the Raman scattering intensity than the hydrophilic micro/nanostructure of ZnO (film)/Ti_3_C_2_T_x_.

### DFT calculation of charge transfer properties

**Fig. 5:**
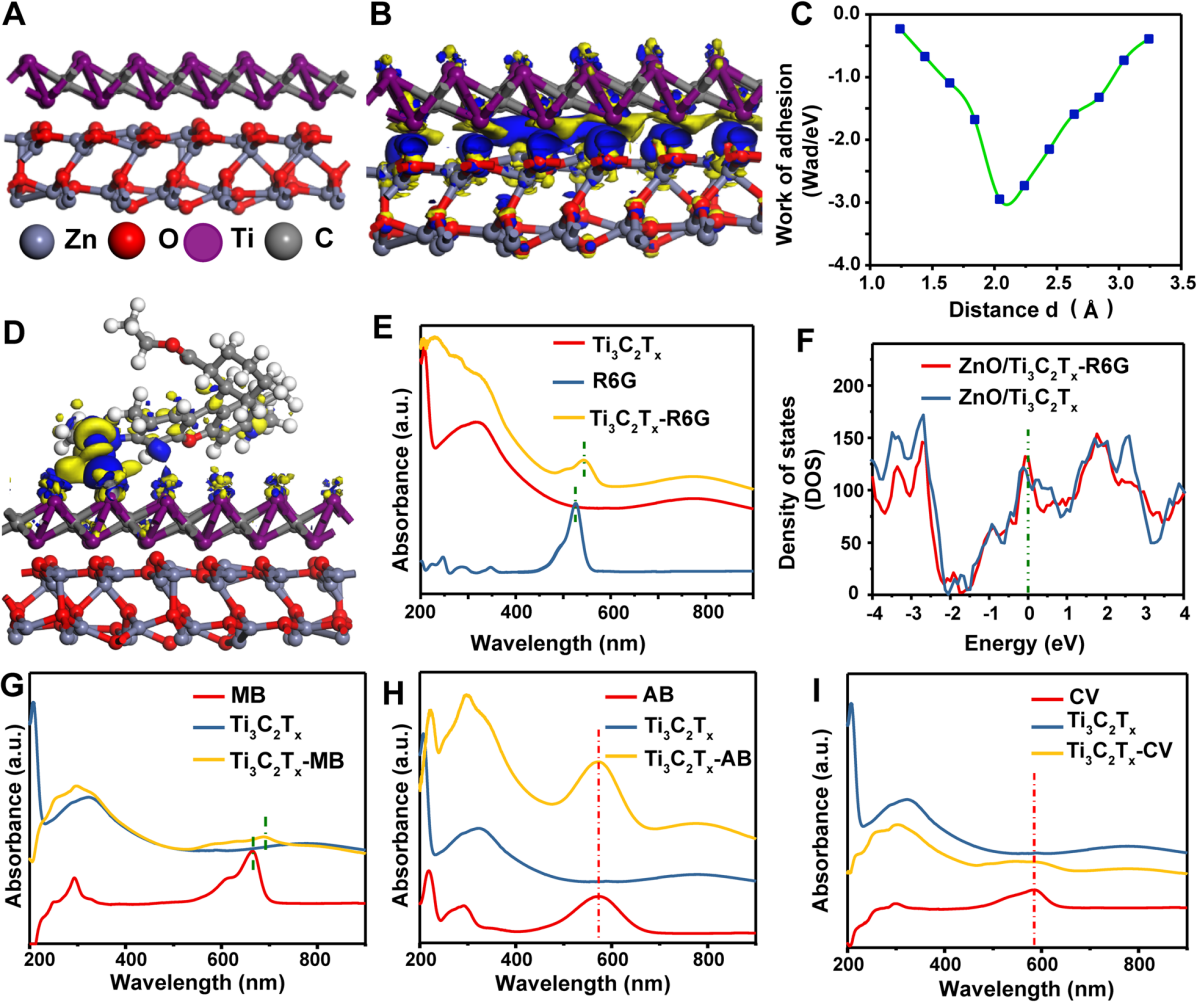
Density functional theory calculations of molecular properties. **A** Optimized interface structure of ZnO/Ti_3_C_2_T_x_. **B** Calculated charge density difference of ZnO/Ti_3_C_2_T_x_. **C** Work of adhesion at the ZnO/Ti_3_C_2_T_x_ interface. **D** Calculated charge density difference of ZnO/Ti_3_C_2_T_x_-R6G. **E** UV–vis spectra of R6G, Ti_3_C_2_T_x_, and Ti_3_C_2_T_x_-R6G measured by Ultraviolet–visible Spectrophotometer. **F** Density of states of ZnO/Ti_3_C_2_T_x_ and ZnO/Ti_3_C_2_T_x_-R6G. **G**–**I** UV–vis spectra of (**G**) MB, Ti_3_C_2_T_x_, and Ti_3_C_2_T_x_-MB, (H) AB, Ti_3_C_2_T_x_, and Ti_3_C_2_T_x_-AB, and (**I**) CV, Ti_3_C_2_T_x_, and Ti_3_C_2_T_x_-CV measured by Ultraviolet–visible Spectrophotometer

To further explore the enhancement mechanism, first-principles DFT calculations were employed. Using the optimized ZnO/Ti_3_C_2_T_x_ (Fig. 5A) and ZnO/Ti_3_C_2_T_x_-R6G (Additional file 1: Figure S5a) interface structures, the charge density difference was calculated. The results clearly identified a charge transfer mechanism at the interface, enabling electron transfer from the ZnO nanorod surface to Ti_3_C_2_T_x_ nanosheet (Fig. 5B). The work of adhesion (W_ad_) describes the relationship between the interface distance and system energy in the interface. The DFT calculations showed that W_ad_ is lowest at an interface distance of ≈2.0 Å (Fig. 5C). This suggests that the distance between ZnO and Ti_3_C_2_T_x_ surfaces is ≈2.0 Å, which results in their chemisorption. The calculated energy of R6G absorption on the ZnO/Ti_3_C_2_T_x_ interface is −2.36 eV, indicating that the interface has a large adsorption capacity for R6G molecules.

DFT simulations were also carried out to investigate charge transfer phenomena at the interface between Ti_3_C_2_T_x_ and R6G and found that charge can be further transferred from Ti_3_C_2_T_x_ to R6G molecules (Fig. 5D, top view Additional file 1: Figure S5b). These results are consistent with UV–vis spectra of Ti_3_C_2_T_x_-R6G (Fig. 5E), which show difference in the peak position between 500 and 550 nm that could be explained by the chemisorption of Ti_3_C_2_T_x_ and R6G. Moreover, the calculated density of states of ZnO/Ti_3_C_2_T_x_-R6G increased toward the Femi level after adsorption of R6G (Fig. 5F), which again indicates there is interfacial charge transfer between the substrate and R6G. Chemisorption was likewise observed between Ti_3_C_2_T_x_ with MB, together with an obvious absorption peak shift (Fig. 5g), suggesting charge transfer also occurs between Ti_3_C_2_T_x_ and MB and leads to high SERS activity (detection limit of 10^−10^ M). However, because of the physisorption of the AB and CV molecules with the Ti_3_C_2_T_x_ surface, no obvious absorption peak shift was observed (Fig. 5H, I), which could explain why the SERS enhancement of AB and CV is limited (Additional file 1: Figure S7b, c). Considering that the group of ZnO(film)/Ti_3_C_2_T_x_ with a larger contact area between Ti_3_C_2_T_x_ and ZnO film did not show more significant enhancement performance, this suggests that the hydrophobic structure plays a more important role in Raman enhancement. These results suggest that the observed SERS performance of the ZnO/Ti_3_C_2_T_x_ substrate may result from a synergy between its analyte-concentrating ability and interfacial charge transfer properties.

**Fig. 6:**
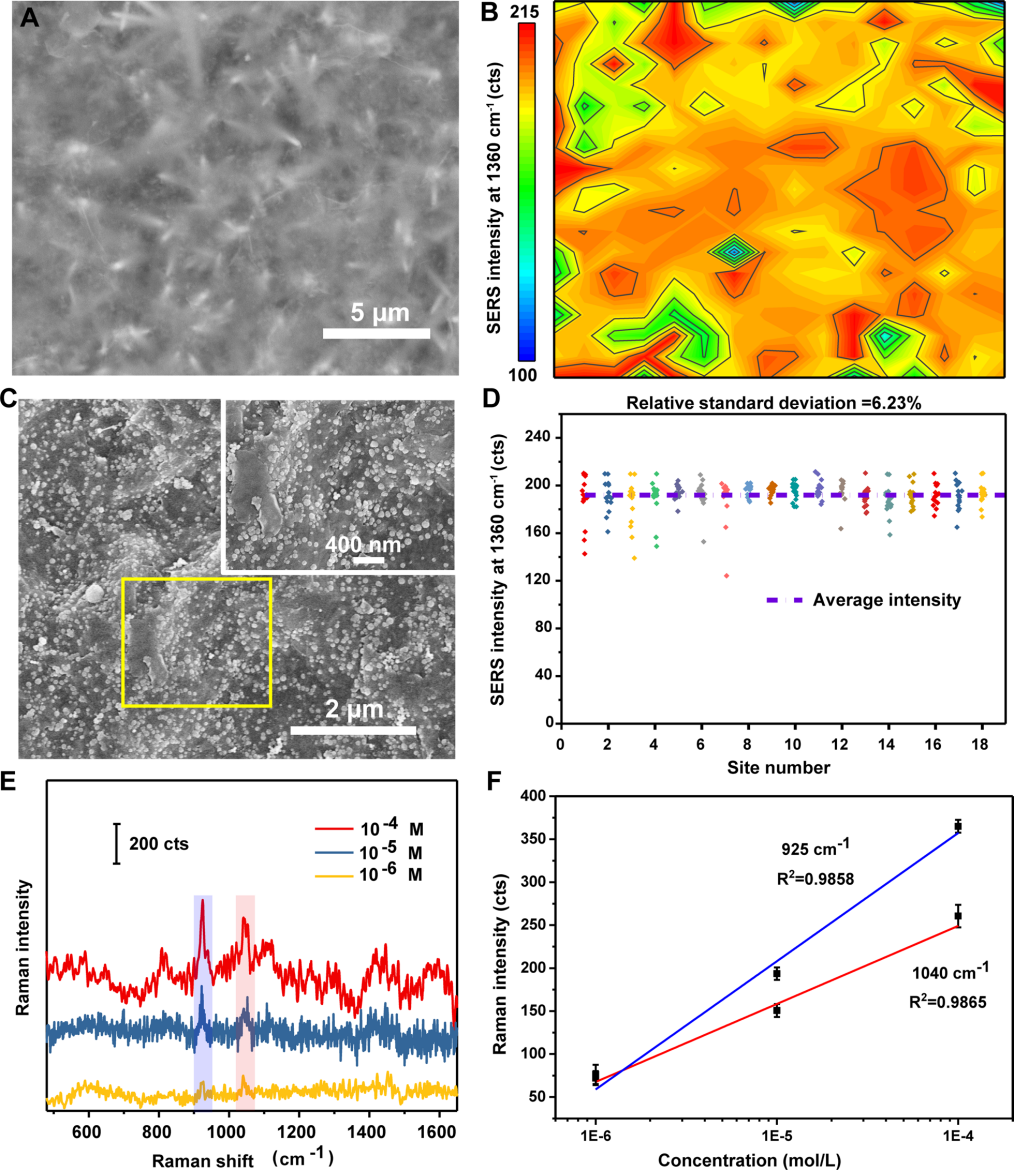
**A** SEM image of the ZnO/Ti_3_C_2_T_x_ substrate. **B** Spatial map of the SERS intensity (at 1360 cm^−1^) of 10^−6^ M R6G drop-cast on ZnO/Ti_3_C_2_T_x_ (n = 16 × 18). **C** SEM images of Au nanoparticles distributed on the ZnO/Ti_3_C_2_T_x_ substrate, inset provides the enlarged image of selected section. **D** The SERS intensity distribution of the 1360 cm^−1^ peak across all mapping sites in panel b. **E** SERS spectra of 10^−4^ to 10^−6^ M microRNA on ZnO/Ti_3_C_2_T_x_. **F** Linear fit plots of peaks of 925 cm^−1^ and 1040 cm.^−1^, respectively (n = 9)

To evaluate the spatial distribution of the SERS intensity, the Raman intensity from 10^−6^ M R6G drop-cast on the ZnO/Ti_3_C_2_T_x_ substrate was mapped at 1360 cm^−1^. The signal distribution on the substrate did not exhibit a coffee ring effect, and its signal was slightly stronger in the depressions than in the protrusions (Fig. 6A and B). To investigate the reason for this phenomenon, we took SEM images of the distribution of gold nanoparticles on the substrate to simulate the distribution of R6G (Fig. 6C). According to the image, gold nanoparticles tend to aggregate more in the depression than elsewhere, so the intensity of the Raman signal in the depression is also slightly stronger than elsewhere. Despite the slightly different molecular distributions, the overall signal intensity remained relatively uniform. Statistical analysis of 1360 cm^−1^ peak intensity in Fig. 6D yielded a relative standard deviation of only 6.23%, which indicates the platform is highly homogeneous and thus capable of providing reproducible signals. Area mapping of 10^–6^ M to 10^–11^ M was performed as can be seen in Additional file 1: Figure S6, with the decrease of analyte concentration, the distribution of Raman signal becomes uneven. This indicates that the distribution of molecules on the substrate is not that uniform with decreasing concentration, which result in the signal variation. A more reasonable sampling method is to first find the location of the analyte by point measurement, and then do the area mapping based on this, then analyze the area where the signal is more concentrated.

Finally, the ability of the ZnO/Ti_3_C_2_T_x_ substrate to detect biological macromolecules was assessed. Using microRNA as an analyte (Fig. 6e), a detection limit of 10^−6^ M was obtained and characteristic peaks at 925 cm^−1^ and 1040 cm^−1^ [45] were clearly observed at microRNA concentrations from 10^−4^ to 10^−6^ M. The measured Raman intensities show a certain linear relationship at the two characteristic peaks of 925 cm^−1^ and 1040 cm^−1^(R^2^_925 cm_^−1^ = 0.9858, R^2^_1040 cm_^−1^ = 0.9865) with a detection limit of 10^–6^ M. We speculate that there are two possible causes of this phenomenon. As can be seen in Additional file 1: Figure S7, the distribution of analytes was uneven at low concentration, which result in the uneven distribution of Raman signal. Another possibility is that different nucleobase pairs (U and A) adsorb differently to the substrate [46–48], resulting in different signal intensities that enhanced by ZnO/Ti_3_C_2_T_x_ substrate. Besides, the ZnO/Ti_3_C_2_T_x_ substrate yielded a much lower Raman intensity than that obtained using a SERS substrate composed of Au nanoparticles (Additional file 1: Figure S8a and b). However, the signal stability of the SERS performance of ZnO/Ti_3_C_2_T_x_ was higher, especially at the peaks of 925 cm^−1^ and 1040 cm^−1^, characteristic peaks can be clearly identified even at 10^–6^ M, which may result from its greater analyte-concentrating ability compared with that of the hydrophilic Au nanoparticles. The stable SERS performance of ZnO/Ti_3_C_2_T_x_ suggests it may provide a new substrate for biological analyte detection.

## Conclusion

In summary, a hydrophobic micro/nanostructured ZnO/Ti_3_C_2_T_x_ SERS substrate was developed and yielded a detection limit of 10^−11^ M using R6G as target analytes. DFT calculations were used to study the mechanism underlying the SERS activity. Synergy between the hydrophobic nanostructure and ZnO/Ti_3_C_2_T_x_ interfacial charge transfer were found to enhance the SERS activity. Moreover, the platform shows potential for miRNA detection. This work provides a new strategy of combining a semiconductor nanoarray with 2D Ti_3_C_2_T_x_ for designing and fabricating high-performance SERS platforms.

## Supplementary Information

**Additional file 1.** Supplementary Material.

## Data Availability

Data availability—the data generated during the current study are available within the article. Supplementary material related to this article can be found in the online version.
