# Supplementary material for: Deposition of hydrophilic Ti3C2Tx on a superhydrophobic ZnO nanorod array for improved surface-enhanced raman scattering performance
Source: J Nanobiotechnology. 2023 Jan 16;21:17. doi: 10.1186/s12951-022-01756-4 (PMC9843901; doi:10.1186/s12951-022-01756-4)
Supplement: Supplementary file 1 — Additional file 1. Supplementary Material. [file 12951_2022_1756_MOESM1_ESM.docx]

Supporting Information

**Deposition of Hydrophilic Ti_3_C_2_T_x_ on a Superhydrophobic ZnO Nanorod Array for Improved Surface-Enhanced Raman Scattering Performance**

Zhihua Wu^a^, De Zhao^a^, Xin Han^c^, Jichang Liu^c^, Ying Sun^a*^, Yaogang Li^b*^, Yourong Duan^a*^

^a^ State Key Laboratory of Oncogenes and Related Genes, Shanghai Cancer Institute, Renji Hospital School of Medicine, Shanghai Jiao Tong University, Shanghai 200032, China

^b^ State Key Laboratory for Modification of Chemical Fibers and Polymer Materials, International Joint Laboratory for Advanced Fiber and Low-Dimension Materials, College of Materials Science and Engineering, Donghua University, Shanghai 201620, China

^c^ State Key Laboratory of Chemical Engineering, School of Chemical Engineering, East China University of Science and Technology, Shanghai 200237, China

* Corresponding authors

E-mail: [yrduan@shsci.org](mailto:yrduan@shsci.org); [yaogang_li@dhu.edu.cn](mailto:yaogang_li@dhu.edu.cn); [ysun@shsci.org](mailto:ysun@shsci.org)

**Calculation of SERS enhancement factors**

Enhancement factors (EFs) was calculated by the following equation:

$EF=\frac{\frac{I_{SERS}}{N_{ads}}}{\frac{I_{bulk}}{N_{bulk}}}$ (1)

where, N_ads_ and N_bulk_ represent the number of R6G molecules in the SERS sample and the control group, respectively. I_SERS_ and I_bulk_ are the same vibration peak of R6G molecule on ZnO/Ti_3_C_2_T_x_ and the normal Raman spectrum from solid sample, respectively. In the experiment, 100 μL of aqueous R6G solution (0.1 M) was dried onto the glass substrate (~ 0.2 × 0.2 cm^-2^) and N_bulk_ can be estimated as:

N_bulk_ = 100 μL × 0.001 mol/L × 6.02 × 10^23^ mol^-1^ × 2 μm^2^ /(0.04 cm^2^) (2)

where the numerical aperture of the objective lens N_A_ = 0.5, thereby, Laser spot size 2 μm^2^. N_bulk_ was estimated to 3.01×10^10^, and the N_ads_ was 2.408×10^4^ (where the concentration is 10^-6^ M, 4 μL).

Table S1. Calculation of EFs at different characteristic peaks

|  | 614 cm^-1^ (×10^6^) | 1360 cm^-1^ (×10^6^) |
| --- | --- | --- |
| ZnO | 5.46 ± 1.90 | 2.98 ± 0.53 |
| Ti_3_C_2_T_x_ | 7.10 ± 0.79 | 4.75 ± 0.42 |
| ZnO /Ti_3_C_2_T_x_ | 14.93 ± 1.24 | 7.88 ± 1.07 |

**Preparation of 50 nm Au nanoparticles**

The Au nanoparticles were prepared as follows. 50 mL deionized water contained 60 µL 0.1 M HAuCl_4_ solution in 200 mL three-neck flask was heated to boil with reflux condenser. Then 350 µL freshly prepared 1% (w/w) Na_3_-citrate aqueous solution was added in the boiling solution and heated for 1 hour. The 50 nm Au nanoparticles were collected through centrifugation for 30 min at 4000 rpm and redispersed in 5 mL deionized water.

**Figures**


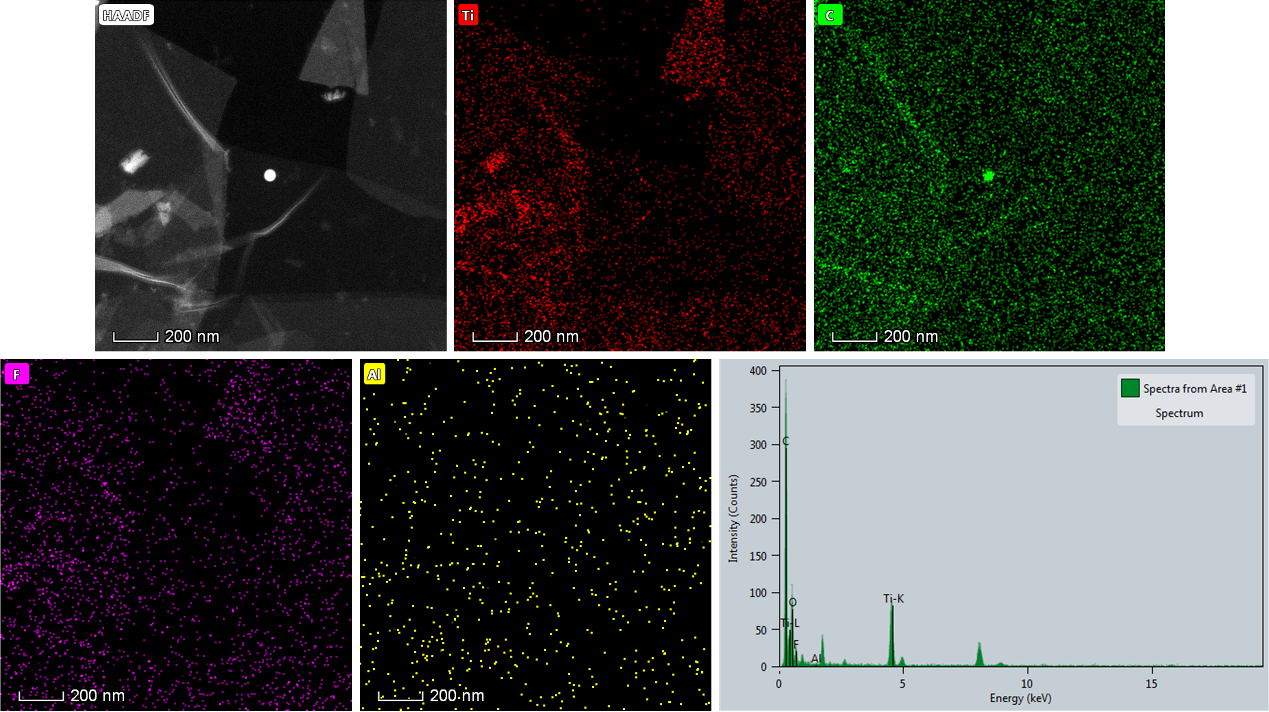


**Figure S1.** X-ray energy dispersive spectroscopy composition corresponding Ti_3_C_2_T_x_ sheets.


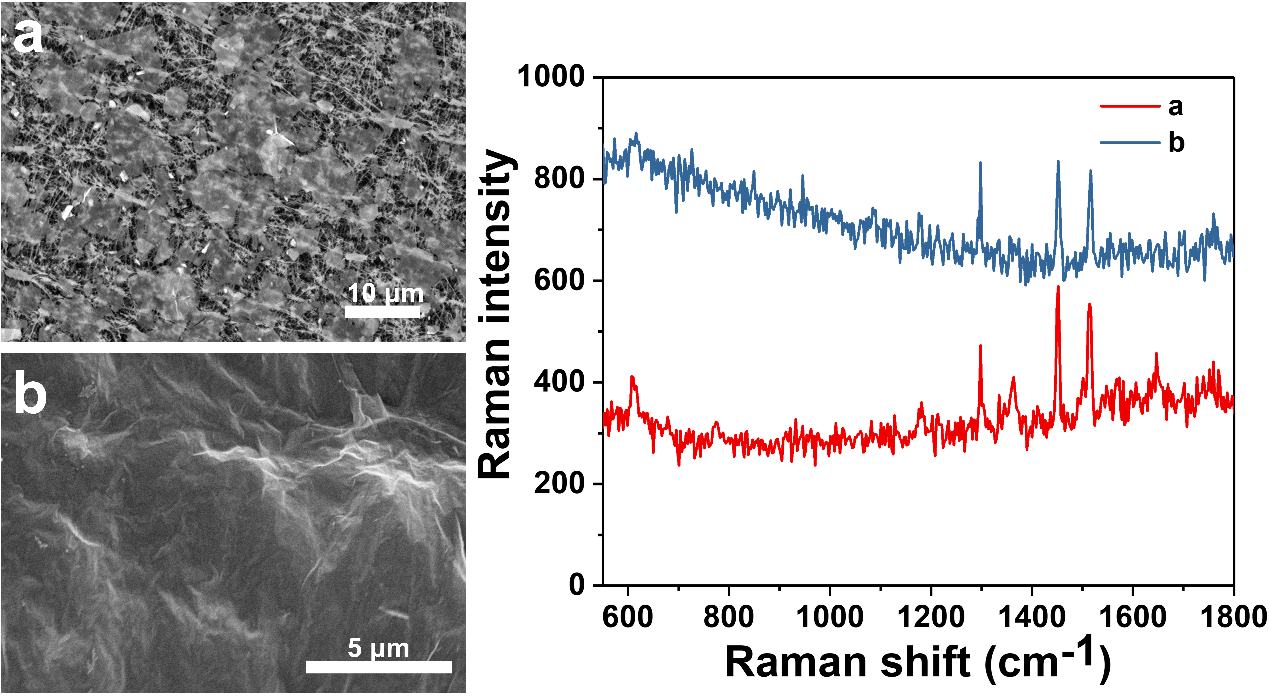


**Figure S2.** Evaluation of different Raman intensities of MXene membranes prepared by different amount of Ti_3_C_2_T_x_ sheets, (a) 0.5 mg/mL, (b) 2mg/mL.


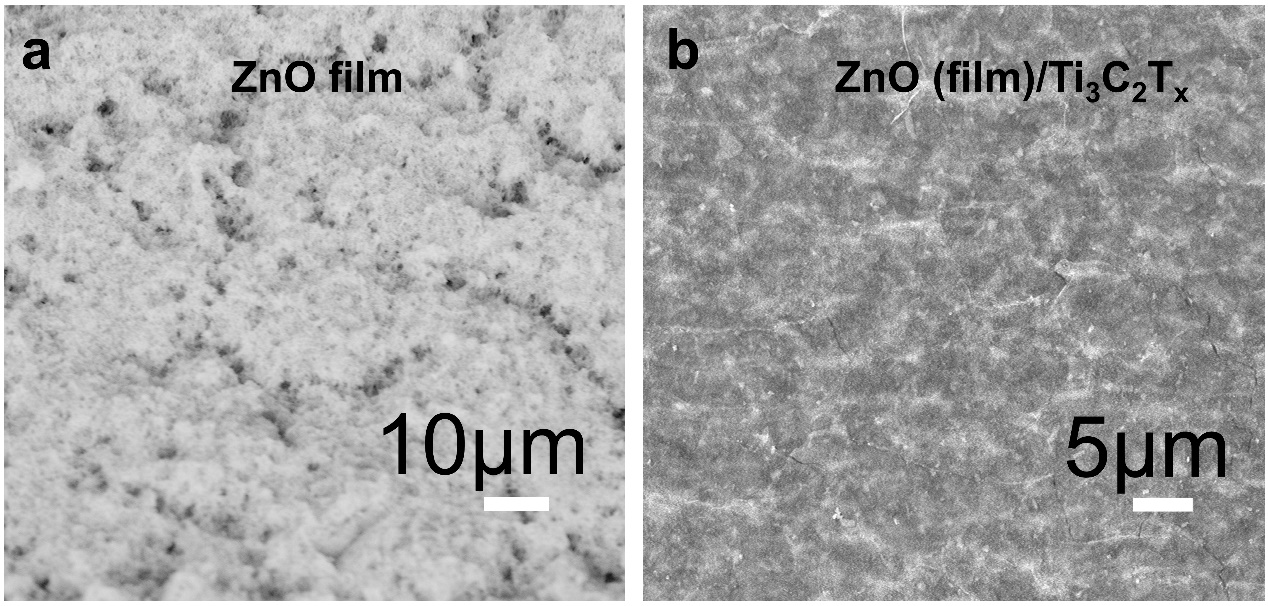


**Figure S3.** SEM images of (a) ZnO film, and (b) ZnO (film)/Ti_3_C_2_T_x_.


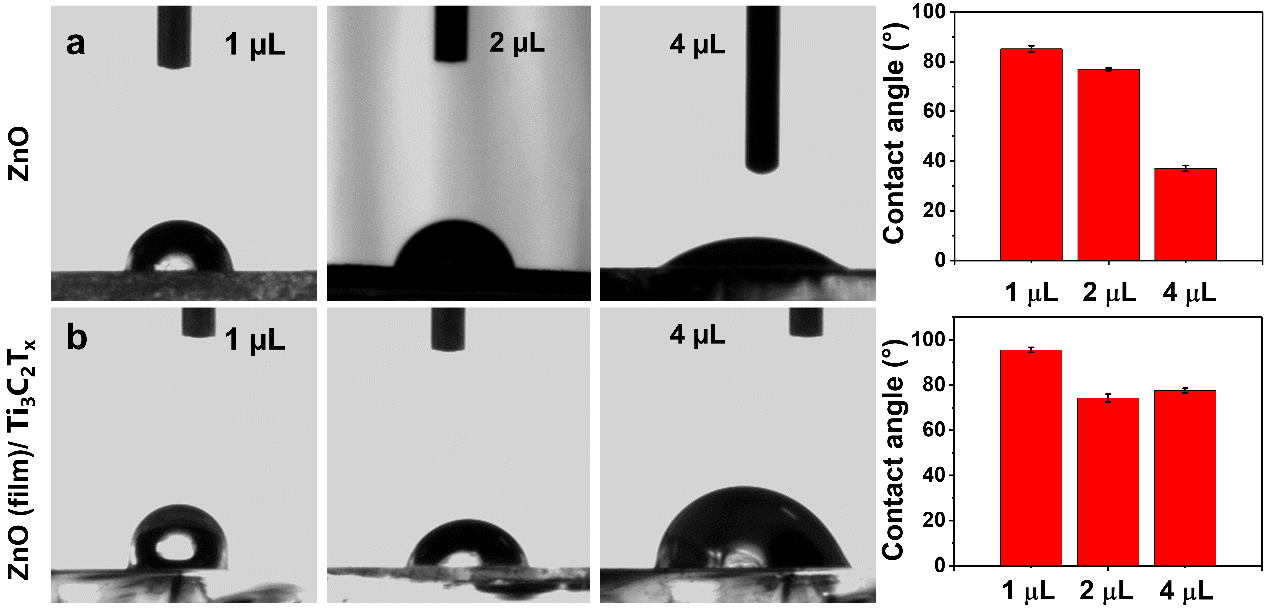


**Figure S4.** Characterization of contact angles of (a) ZnO films, (b) ZnO (film)/Ti_3_C_2_T_x_ SERS substrate.


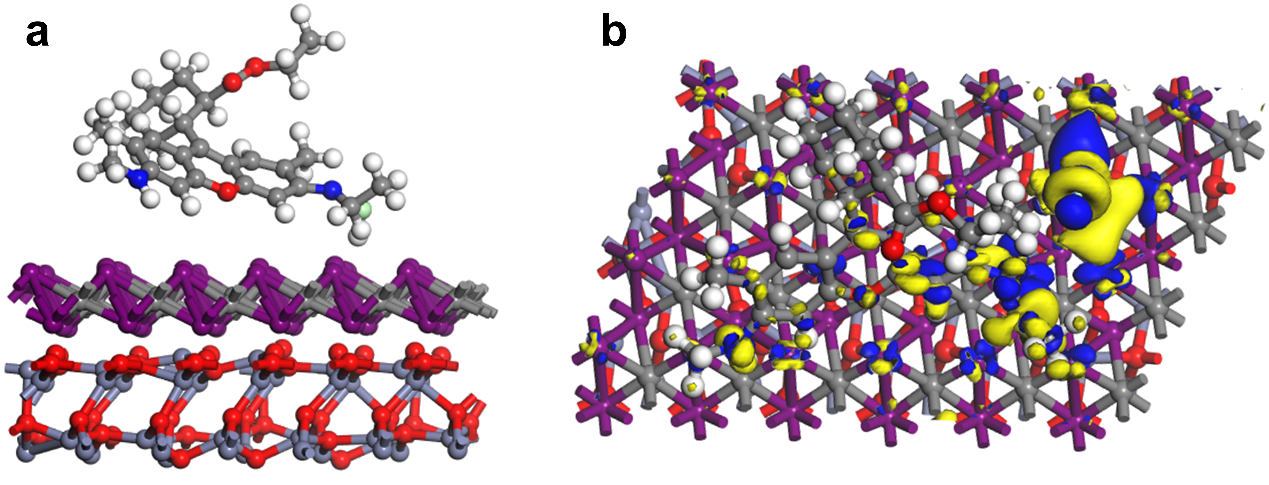


**Figure S5.** The charge density of R6G molecules adsorbed on the Ti_3_C_2_T_x_ surface, (a) R6G molecules adsorbed on the ZnO/Ti_3_C_2_T_x_ surface, (b) top view of the calculated charge density difference.


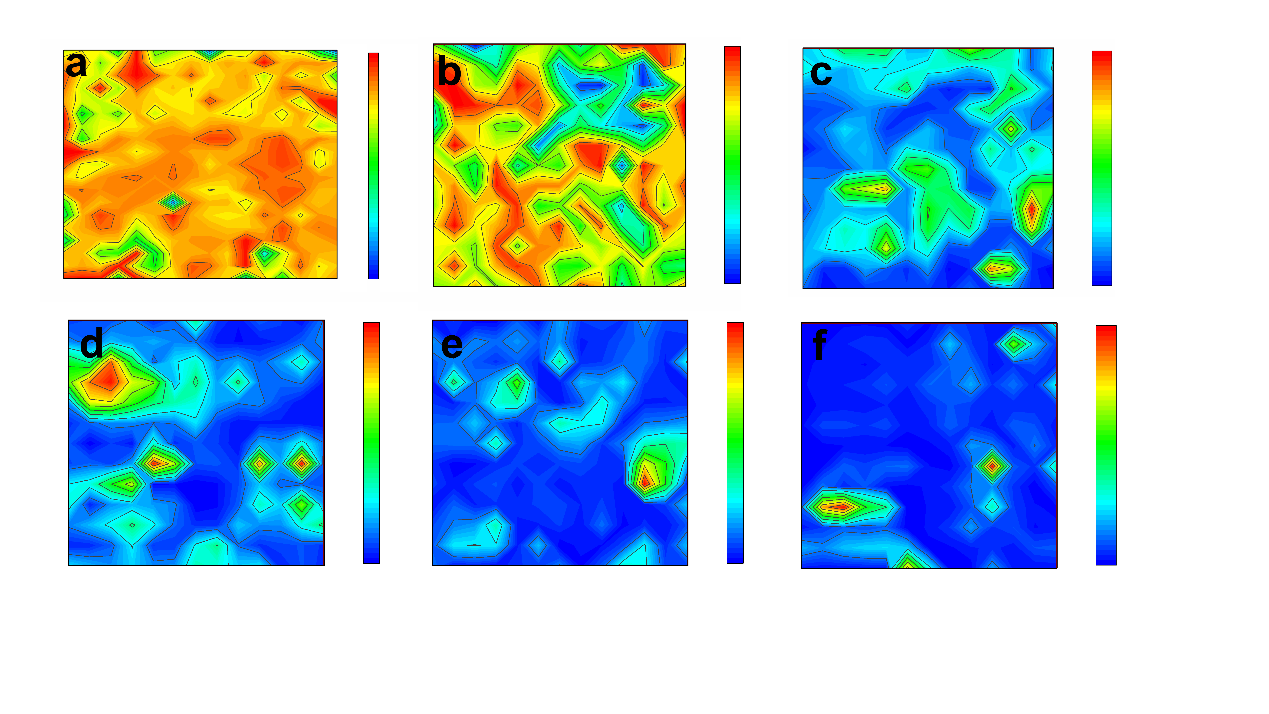


Fig. S6 Spatial map of the SERS intensity of (a) 10^−6^ M, (b) 10^-7^ M, (c) 10^-8^M, (d) 10^-9^M, (e) 10^-10^M, (f) 10^-11^M R6G drop-cast on ZnO/Ti_3_C_2_T_x_.


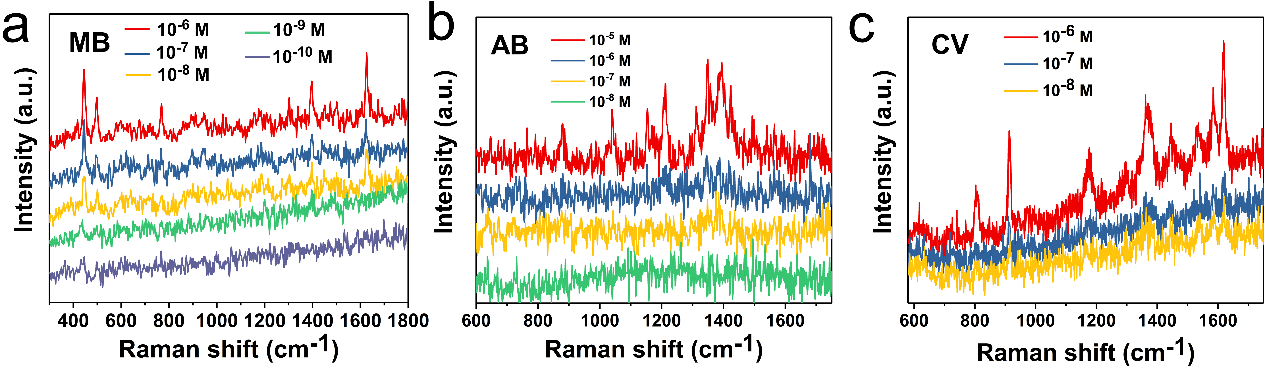


**Figure S7.** Raman intensities of different organic molecule by ZnO/Ti_3_C_2_T_x_ substrate, (a) MB, (b) AB, (c) CV.


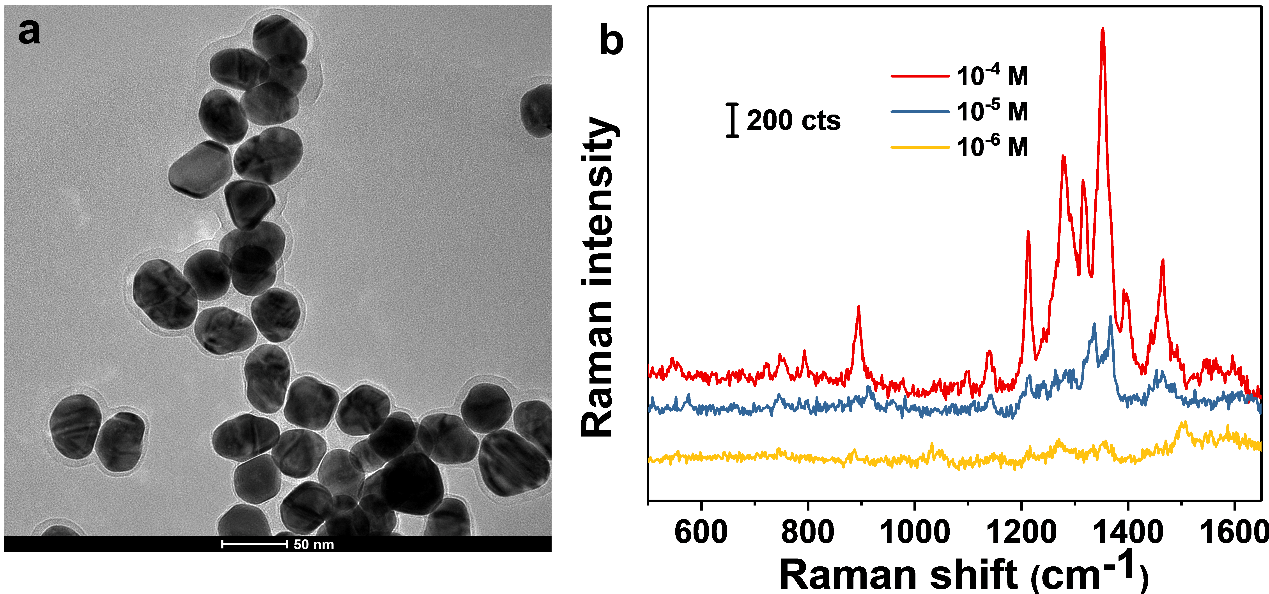


**Figure S8.** (a) TEM of prepared Au nanoparticles and (b) Raman spectra of miRNA enhanced by Au nanoparticles.
